# Post-Operative Treatment of Abdominal Section in Women1Read before the Gynecological Section of the American Medical Association, June 8, 1900.

**Published:** 1900-08

**Authors:** Walter B. Chase

**Affiliations:** Borough of Brooklyn, New York City, 263 Hancock Street


### POST-OPERATIVE TREATMENT OF ABDOMINAL SECTION IN WOMEN.[1]

By WALTER B. CHASE, M. D., Borough of Brooklyn, New York City.

*(Author's Abstract.)*

THE author said that, other things being equal, the best after-results in these cases will follow the ones in which proper preparation had been made, and the operation performed in keeping with modern aseptic surgery.

In closing the abdominal incision, great care in coaptation, the use of chromicised catgut for layer suturing and silkworm gut for the through and through method were recommended. Sutures must not be tied too tight, or allowed to remain in too short a time, the writer recommending that in from ten to twenty days alternate sutures be removed, and a few days later the remaining ones. He condemned

1. Read before the Gynecological Section of the American Medical Association, June 8, 1900.

the employment of buried unabsorbable sutures, as they remain as foreign bodies, liable to cause pain and ulceration and therefore were unscientific and contrary to sound surgical procedure.

Unless for fear of hemorrhage or undue traction he recommended greater latitude of position after laparatomy than has usually been permitted, as contributing to the patient's comfort and the smoothness of convalescence.

For shock he advised, before the close of the operation, a high enema of hot salt solution with an ounce of whiskey, together with proper cardiac stimulants, and, if necessary placing the head lower than the trunk.

In hemorrhage he advised promptness in arresting the bleeding and in emergency to operate on the bed without moving the patient. He said that while "blood is thicker than water, water is better than no blood," and that intravenous or intracellular injection of salt solution forms a most brilliant chapter in conservative surgery.

As for drainage he declared that every case must be a law unto itself, and he took issue with the statement that drainage means imperfect surgery, saying that the statement is illogical and dangerous, although he admits that the improved technique of today made the need of drainage less frequent. The results obtained in cases of septic peritonitis by through and through drainage was commented upon with hopeful interest.

The author stated that flatulence, nausea and vomiting were often unavoidable. If due to ether narcosis it would subside spontaneously. For nausea, he advocated frequent drachm doses of hot water, and for persistent vomiting high enemas of warm water containing two or three drachms of saturated solution of magnesium sulphate until the bowels were opened.

Nutrition should be administered cautiously, beginning twelve hours after the operation and should be entirely liquid, consisting at first of broth, then of beefsteak juice and limewater with milk.

He said that close watch must be kept of the urine both as to quantity and quality, as showing possible injury to the urinary tract and also diminution or suppression of the renal secretion. Suppression, partial or entire, must be energetically treated by salines, pure water by mouth, salt solution by the rectum, and if arterial pressure is diminished the use of digitalis and persistent dry cupping.

*Pulse.*—As a guide to diagnosis, prognosis and treatment in pelvo-abdominal surgery, he recommends careful study of the pulse as suggestive of complications. As is the pulse so is the patient. The preoperative pulse must be taken as the standard of comparison. Usually in cases which do well the pulse falls within the first twenty-four hours; while increasing rapidity suggests troublesome complications.

*Temperature.*—Attention was called to the fact that closely allied with the causes which induce rapid pulse are those which manifest themselves in connection with the use of temperature. It may be safely assumed that a proper appreciation of the causes which lead to variation of pulse and temperature will be the key to their management, whether the fever arise from sepsis, suppuration, peritonitis, malaria or other agencies, its course must be differentiated and the appropriate treatment applied.

He emphasised as a necessity that pus within the pelvic cavity must be evacuated and drainage established. So also the stitches in stitchhole abscesses must be removed and pus pockets disinfected. The writer affirmed that temperature arising form septic processes, either local or systemic, are usually the gravest complications which confront the operator.

The rapid pulse and feeble cardiac contractions with low fever or subnormal temperature must be distinguished from that attending depression and slow exhaustion. So also the advent of intestinal perforation or the evacuation of pus into the peritoneal cavity must be recognised and treated. Hyperpyrexia, due to intraperitoneal infection demands the prompt use of saline catharsis. The value of heat abstraction under such conditions by the use of the head and abdominal coil has its uses and limitations.

*Pain.*—The pain following laparatomy clamors for relief and cannot be altogether ignored, but it must not, the author insists, be inferred that it always or usually requires the use of anodynes. In fact he frequently finds them required—and when indicated usually restricts himself to codein, as it is less purturbing in its after-effects and interferes much less with peristalsis and secretions as compared with morphia.

The exhaustion following laparatomy may be of serious moment. Here the writer recommends prudent and well sustained alimentation, stimulation with supporting tonics, the most careful attention to good nursing, and the best hygienic surroundings.

263 HANCOCK STREET.

